# Photostability and Performance of Polystyrene Films Containing 1,2,4-Triazole-3-thiol Ring System Schiff Bases

**DOI:** 10.3390/molecules21121699

**Published:** 2016-12-09

**Authors:** Gassan Q. Ali, Gamal A. El-Hiti, Ivan Hameed R. Tomi, Raghad Haddad, Alaa J. Al-Qaisi, Emad Yousif

**Affiliations:** 1 Department of Chemistry, College of Science, University of Al-Mustansiriyah, Baghdad 10052, Iraq; qgassan@gmail.com (G.Q.A.); ivanhrtomy@gmail.com (I.H.R.T.); 2 Cornea Research Chair, Department of Optometry, College of Applied Medical Sciences, King Saud University, P.O. Box 10219, Riyadh 11433, Saudi Arabia; 3 College of Pharmacy, Privet Uruk University, Baghdad 10069, Iraq; raghadalihaddad@gmail.com; 4 Department of Chemistry, College of Science, Al-Nahrain University, Baghdad 64021, Iraq; alaaalqaisisma@gmail.com

**Keywords:** photostabilization, UV light, polystyrene, 1,2,4-triazole-3-thiol, functional group index, Schiff bases

## Abstract

Series of 4-(4-substituted benzylideneamino)-5-(3,4,5-trimethoxyphenyl)-4*H*-1,2,4-triazole-3-thiols were synthesized and their structures were confirmed. The synthesized Schiff bases were used as photostabilizers for polystyrene against photodegradation. Polystyrene polymeric films containing synthesized Schiff bases (0.5% by weight) were irradiated (λ_max_ = 365 nm and light intensity = 6.43 × 10^−9^ ein·dm^−3^·s^−1^) at room temperature. The photostabilization effect of 1,2,4-triazole-3-thiols Schiff bases was determined using various methods. All the additives used enhanced the photostability of polystyrene films against irradiation compared with the result obtained in the absence of Schiff base. The Schiff bases can act as photostabilizers for polystyrene through the direct absorption of UV radiation and/or radical scavengers.

## 1. Introduction

Polystyrene (PS) is an aromatic synthetic polymer that is produced in millions of tons every year. It was reported that 55 million tons of polystyrene was produced in 2013 [1]. It is a widely used plastic produced from polymerization of styrene. Various types of polystyrene are known that mainly depend on the positions of phenyl groups along the polymeric chain [2,3,4]. Polystyrene is naturally colorless and transparent and can be used in the manufacture of containers, bottles, electronics, and insulation [2].

One of the main problems associated with the use of polymeric materials, either synthetic or semisynthetic, is their photodegradation [5]. Polystyrene undergoes photodegradation when exposed to harsh environments such as high temperatures and sunlight [6]. Polystyrene is not biodegradable, meaning that it will undergo very slow degradation which may require many years [7]. Photodegradation of polystyrene can lead to chain scission, cross-linking, and discoloration [8]. Therefore, polystyrene polymeric materials should be stabilized against photodegradation and photo-oxidation to prevent and/or reduce the effect weathering conditions. In addition, stabilization would maximize the long-term use of polystyrene to increase its economic viability. Various additives have been used to enhance the photostability of polymeric films such as plasticizers [9], heterocycles [10,11,12,13], aromatics [14,15,16], and organometallics [17,18,19]. Such photostabilizers in most cases can act as direct UV absorbers, radical scavengers, excited state quenchers and/or peroxide decomposers [8].

Recently, we have reported successful polymeric films photostabilization processes by the use of various additives at low concentrations [20,21,22,23,24,25] as part of our continuing interest in the field of polymeric materials [26,27,28,29,30]. Now, we report the photostabilization of polystyrene films in the presence of 1,2,4-triazole-3-thiol ring system Schiff bases.

## 2. Results and Discussion

### 2.1. Synthesis of Schiff Bases ***1**–**4***

**Scheme 1:**
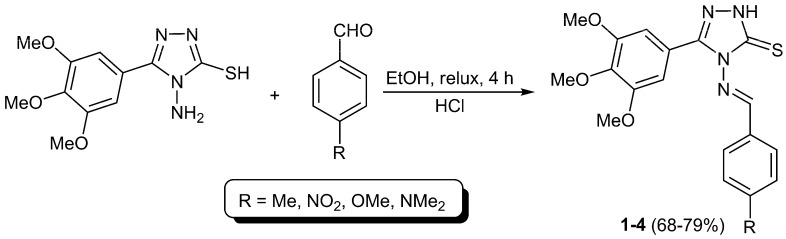
Synthesis of 4-(4-substituted benzylideneamino)-5-(3,4,5-trimethoxyphenyl)-4*H*-1,2,4-triazole-3-thiols **1**–**4**.

4-(4-Substituted benzylideneamino)-5-(3,4,5-trimethoxyphenyl)-4*H*-1,2,4-triazole-3-thiols **1**–**4** were synthesized in 68%–79% yields (Table 1) from reaction of 4-amino-5-(3,4,5-trimethoxyphenyl)-4*H*-1,2,4-triazole-3-thiol and various aromatic aldehydes (4-methylbenzaldehyde, 4-nitrobenzaldehyde, 4-methoxybenzaldehyde, and 4-(dimethylamino)benzaldehyde) in anhydrous ethanol in the presence of few drops of hydrochloric acid as a catalyst under reflux for 4 h (Scheme 1).

### 2.2. Characterization of Schiff Bases ***1**–**4***

The structures of Schiff bases **1**–**4** have been confirmed by the IR and ^1^H-NMR spectral data. The FT-IR spectra showed characteristics stretching vibration bands for the CH=N bonds that resonate within the 1589–1614 cm^−1^ region. Also, they showed absorption bands at the 1240–1244 cm^−1^ region corresponding to the C=S bonds. Moreover, the FT-IR spectra of **1**–**4** showed the absence of the NH_2_ streching bands for aminotriazole or the C=O bands for aromatic aldehydes [31]. Some of the most common and abundant absorption bands for Schiff bases **1**–**4** are represented in Table 1.

The ^1^H-NMR spectra for **1**–**4** confirmed the presence of two different types of aromatic protons. They show two doublets (two protons each; *J* = 8.1–8.4 Hz) that resonated within the 7.73–8.30 ppm region, corresponding the aromatic protons from the aldehyde moeity. They also showed singlet signals (two protons) that are corresponding to the other aromatic protons (7.20–7.24 ppm), from the amine moeity. Also, they show singlet signals that resonate in the 9.56–9.13 ppm region due to the CH proton. This is a clear indication for the formation of the Schiff bases **1**–**4**. However, the SH proton was only apparent in compound **4** and resonates as an exchangeable singlet signal at 14.09 ppm. The ^1^H-NMR spectral data for **1**–**4** are shown in Table 2.

### 2.3. Measuring Photostabilization of Polystyrene Films by IR Spectroscopy

**Figure 1:**
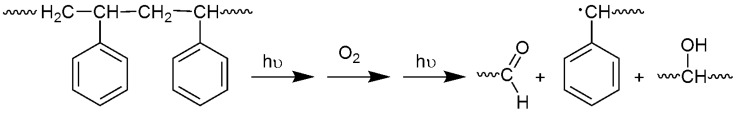
Photooxidative degradation of PS.

**Figure 2:**
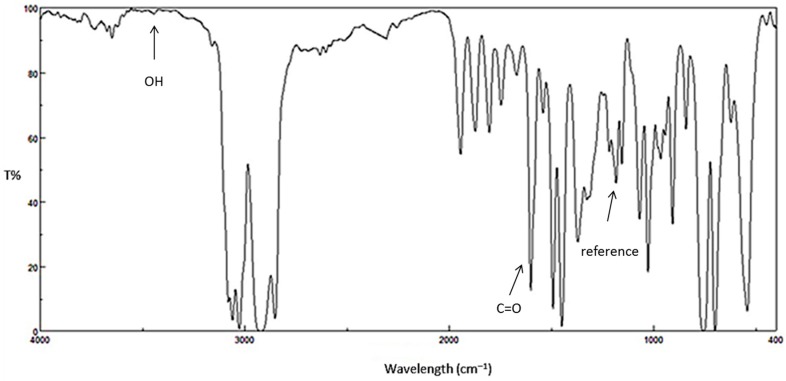
FTIR spectrum for PS film before irradiation.

**Figure 3:**
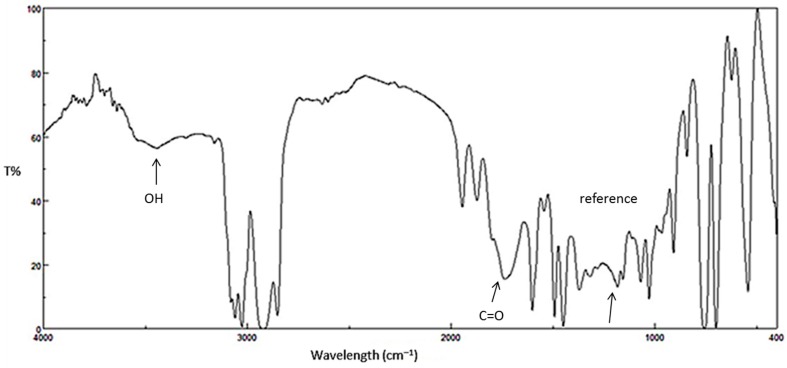
FTIR spectrum for PS film after irradiation (250 h).

Ultraviolet radiation has harmful effects on polystyrene that lead to chemical changes within the polymeric chains. As a result, the polymeric materials could lose their mechanical properties and become discolored [32]. Photo-oxidation of polystyrene leads to the formation of several functional groups [33,34]. It has been reported that irradiation of polystyrene in the presence of oxygen can lead to the production of carbonyl and hydroxyl group moieties, for example, as shown in Figure 1 [35]. Initial degradation of PS leads to a polystyrene radical [–CH_2_CH(Ph)]. Such radicals can abstract a proton from other polymeric chains to produce various other radicals [–CH_2_C(Ph)–CH_2_–, –CH_2_C(Ph)–CH_3_ and –CH_2_CHC(H)–CH(Ph)–]. Theses radicals react with oxygen to produce the corresponding peroxy radicals [8]. Therefore, the changes in the IR spectrum of polystyrene due to irradiation can be used as a measure of photodegradation within the polymeric materials. The FT-IR spectra for PS films before and after irradiation (250 h) by UV light (λ_max_ = 365 nm) are represented in Figure 2 and Figure 3, respectively.

**Figure 4:**
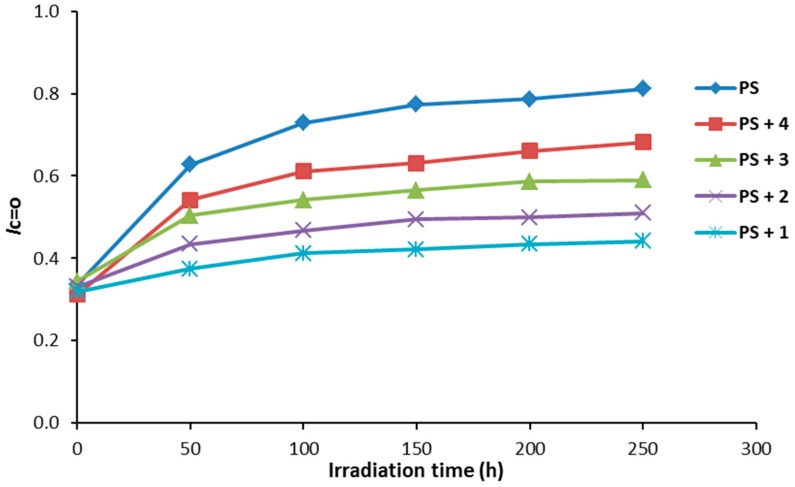
The effect of irradiation on the *I*_CO_ index for PS films.

**Figure 5:**
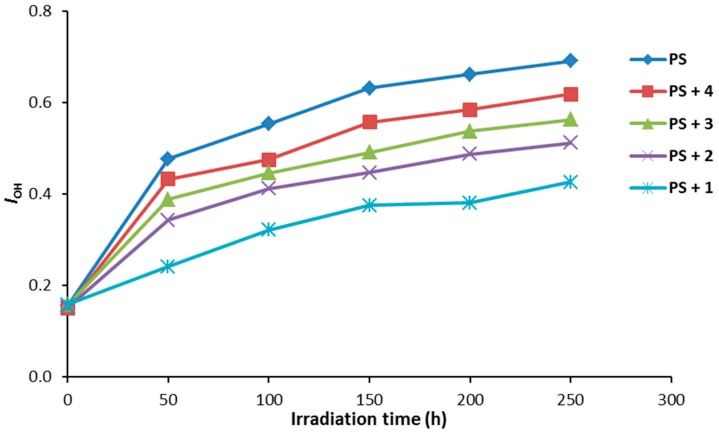
The effect of irradiation on the *I*_OH_ index for PS films.

Compounds **1**–**4** (0.5% by weight) were mixed with PS to produce the polymeric films (40 μm thickness). The efficiency of the additives as photostabilizers for the photostabilization of PS films was investigated under irradiation for 250 h. The PS films were irradiated for 250 h and the carbonyl (*I*_CO_) and hydroxyl (*I*_OH_) indices were monitored using an IR spectrophotometer. The IR absorption bands that appeared at ca. 1720 and 3400 cm^−1^ can be assigned to the carbonyl and hydroxyl groups, respectively [36]. The increases in both *I*_CO_ and *I*_OH_ indexes, compared to the reference peak (1328 cm^−1^) can be used as an indicator for PS photodegradation. It should be noted that *I*_C=O_ does not starts from zero because some photodegradation takes place during the preparation the PS films. The changes in the *I*_C=O_ and *I*_OH_ indices on irradiation of PS in the presence of Schiff bases **1**–**4** are represented in Figure 4 and Figure 5, respectively.

As demonstrated through our findings, all the additives used stabilized the PS film against photodegradation. The changes in both the *I*_CO_ and *I*_OH_ indices were lower for the PS films containing the additives **1**–**4** compared to the ones for the PS (blank). Compound **1** was the most efficient additive for the photostabilization of PS film.

### 2.4. Measuring Photostabilization of Polystyrene Films by Weight Loss

**Figure 6:**
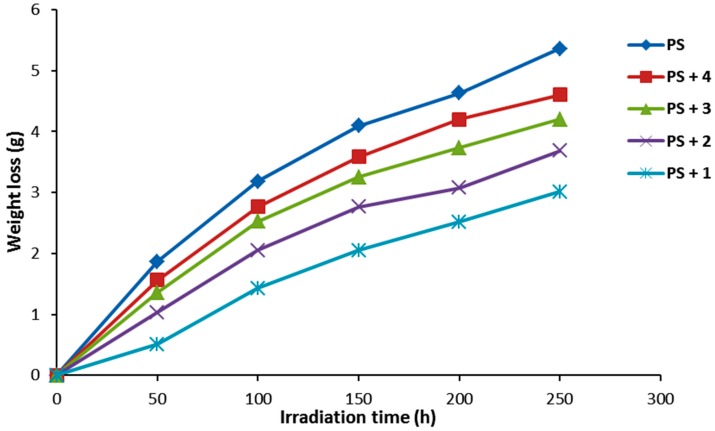
The effect of irradiation on weight loss for PS films.

Photodegradation of PS produces low molecular weight fragments along with volatiles and leads to weight loss [37]. The efficiency of additive (0.5% by weight) photostabilizer for PS was determined in terms of PS weight loss under irradiation for 250 h. The effect of irradiation on the weight loss of PS films is represented in Figure 6. The PS film weight loss increases gradually with the irradiation time. Evidently, the weight loss was low for the PS films containing compounds **1**–**4** compared to that obtained for the PS film (blank) in the absence of such additives. The weight loss was lowest when compound **1** was used as the additive.

### 2.5. Measuring Photostabilization of Polystyrene Films by Variation in Molecular Weight

**Figure 7:**
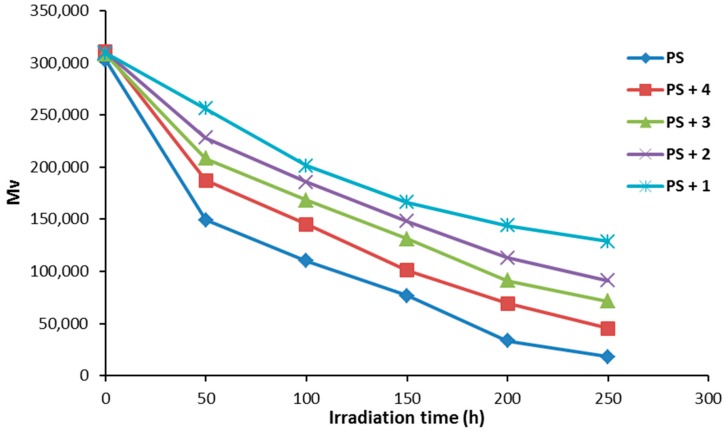
The effect of irradiation on the viscosity average molecular weight for PS films.

The variation of PS molecular weight during photolysis was investigated. Figure 7 shows the relative changes in the average molecular weight (${\bar{M}}_{V}$) for PS films (40 μm thickness) in the presence and absence of additives **1**–**4** (0.5% by weight). The decrease in ${\bar{M}}_{V}$ for the PS films was sharp during the first 50 h and less noticeable thereafter. Photodegradation of PS films leads to a reduction in viscosity due to the formation of degraded polymeric chains of low molecular weight [38]. All additives were efficient against the photodegradation of PS films, compared to the PS films (blank) without additives. Compound **1** was once again the most efficient at photostabilizing the PS films.

The measurements of the initial viscosity average molecular weight (${\bar{M}}_{V,O}$) and a specific irradiation time (${\bar{M}}_{V,t}$) will allow the calculation of the average number of the chain scissions (*S*) as shown in Equation (1) [38].

(1) $$S={\bar{M}}_{V,O}/{\bar{M}}_{V,t}-1$$

**Figure 8:**
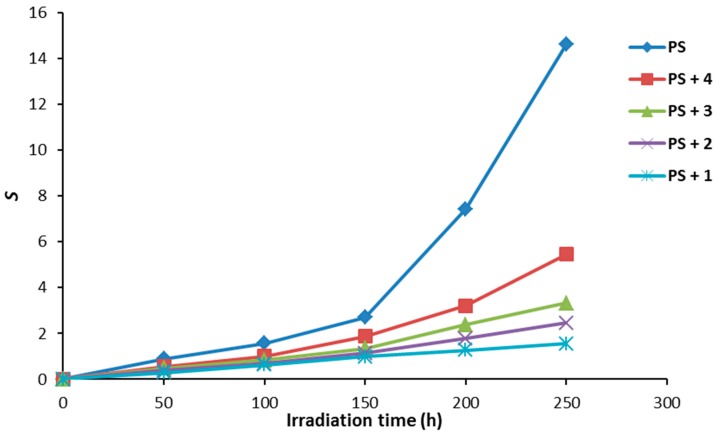
The effect of irradiation on the main chain scissions (*S*) for PS films.

Figure 8 shows the effect of irradiation time on the *S* values. Irradiation of PS (blank) showed a higher degree cross-linking and/or branching compared to the PS films in the presence of additives **1**–**4**. There was a sharp increase in the value of *S* for control PS between 150 and 250 h. Also, significant insoluble residues being formed during the irradiation process are an indication for polymeric chains crosslinking. On the other hand, the increase in the S value for PS containing Schiff base **1** was negligible compared to the others and low proportion of insoluble polymeric materials were observed.

The degree of deterioration (α) for PS films provides a measure for the rapid break of randomly distributed weak bonds at the initial stage of photodegradation. Equation (2) can be used to calculate the α value that depends on the viscosity average molecular weight (${\bar{M}}_{V}$), chain scissions (*S*), and molecular weight (*m*).

(2) $$\alpha =m\times S/{\bar{M}}_{V}$$

**Figure 9:**
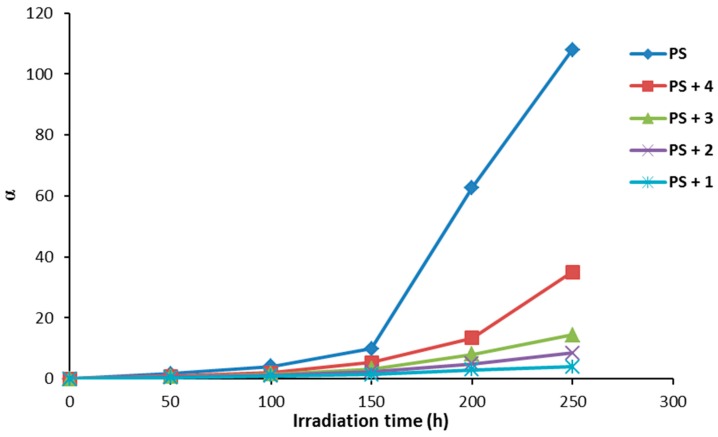
The effect of irradiation on the degree of deterioration (*α*) for PS films

Figure 9 shows the effect of irradiation time on the degree of deterioration (α). The α values were very high for the PS films (blank) on irradiation compared to the samples containing additives **1**–**4**. There was a sharp increase in the value of *α* when the irradiation time was between 150–250 h. The α values for PS films containing additives were very low compared to PS (blank). The α values were minimal for PS films in the presence of Schiff base **1** under irradiation.

The degree of polymerization (*DP*) is the number of monomeric units in a homopolymer. It can be calculated using Equation (3) based on the average molecular weight (*M*_n_) and molecular weight of the monomer unit (*M*_0_) [38,39].

(3) $$DP_{n}=X_{n}=M_{n}/M_{0}$$

**Figure 10:**
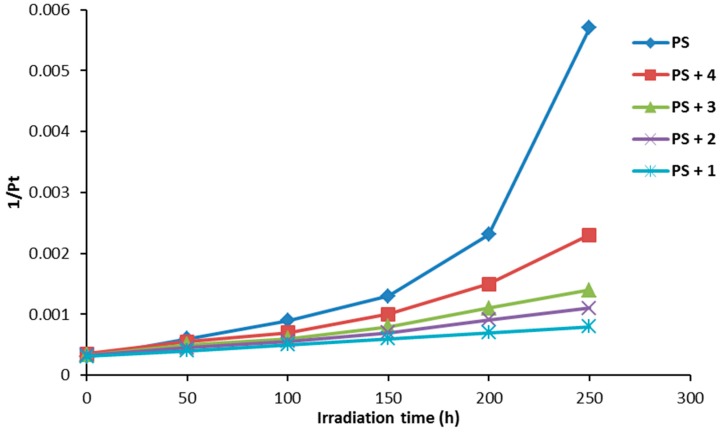
The effect of irradiation on the average degree of polymerization (1/*Pt*) for PS films.

Figure 10 shows the effect of irradiation on the reciprocal degree of polymerization (1/*Pt*). The curve indicates a sharp increase in 1/*Pt* with irradiation time for PS film (blank) compared to the ones obtained in the presence of additives **1**–**4**. The changes in 1/*Pt* were very low when compound **1** (0.5% by weight) was mixed with the PS.

### 2.6. Photostabilization of Polystyrene Films Suggested Mechanisms

**Scheme 2:**
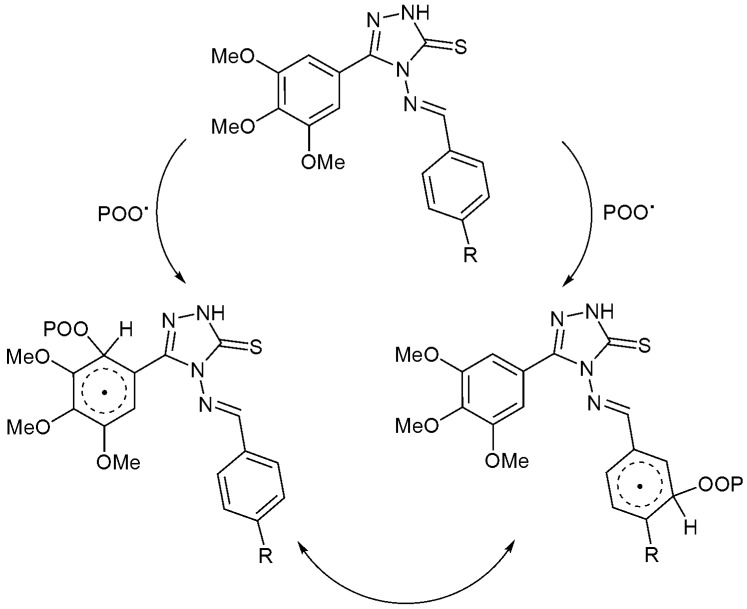
Additives **1**–**4** act as radical scavengers.

Schiff bases contain heterocycles and aryl moieties, in the presence of a chromophore (POO**·**), that could stabilize the PS samples by acting as radical scavengers. A complexation between the additive and the chromophore could be achieved in an excited state in which the energy can be transferred. The resonance within the aryl moieties could stabilize the unreactive charge transfer complexes to a level that is harmless to the polymeric chains (Scheme 2).

**Scheme 3:**
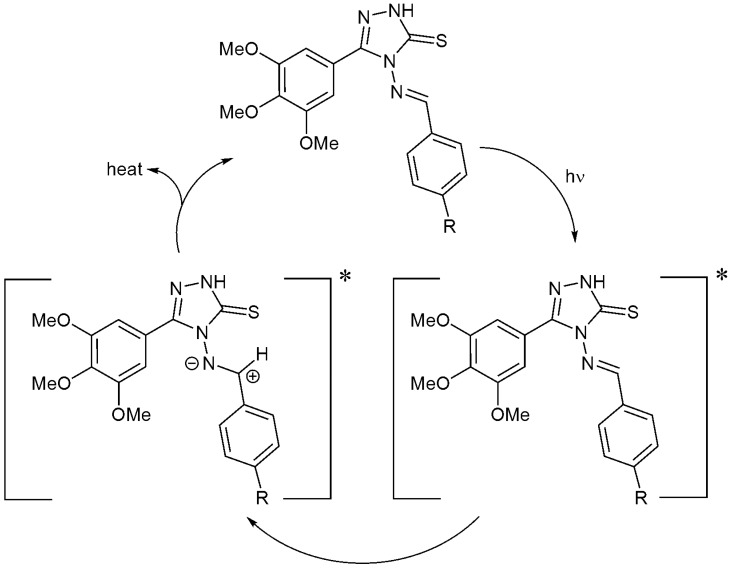
Additives **1**–**4** act as UV absorbers.

Heterocyclic and aryl moieties of the additives embedded within the polystyrene polymeric chains can absorb UV light directly [8]. Schiff bases **1**–**4** have both aryl and triazole ring systems that can act as UV absorbers. They can absorb the harmful UV light and convert it into heat that is harmless to polystyrene (Scheme 3).

## 3. Experimental Section

### 3.1. General

Chemicals and reagents were obtained from BDH Chemicals (Poole, UK) and Sigma-Aldrich Chemical Company (Gillingham, UK). Melting points were recorded on a hot stage Gallenkamp melting point apparatus. The ^1^H-NMR spectra (300 MHz) were recorded on Bruker Ultrashield (Bruker, Coventry, UK) in DMSO-*d*_6_ with tetramethylsilane as an internal standard.

### 3.2. Synthesis of Schiff Bases ***1**–**4***

A mixture of 4-amino-5-(3,4,5-trimethoxiyphenyl)-1,2,4-triazole-2-thiol [40] (0.2 g, 0.7 mmol) and appropriate aromatic aldehyde (0.07 mmol) in absolute ethanol (10 mL) containing one drop of concentrated hydrochloric acid was refluxed for 4 h. The solid obtained on cooling was filtered, washed with hot ethanol, and dried to give Schiff bases **1**–**4** in 68%–79% yields.

### 3.3. Films Preparation

A mixture of polystyrene and Schiff bases was dissolved in chloroform and the films were prepared using the evaporation technique at 25 °C. A Digital Vernier Caliper 2610A micrometer (Vogel GmbH, Kevelaer, Germany) was used to fix the thickness of PS films as 40 μm.

### 3.4. Accelerated Testing Technique

The PS films were irradiated with UV light (λ_max_ = 365 nm and light intensity = 6.43 × 10^−9^ ein·dm^−3^·s^−^^1^) at room temperature using an accelerated weather-meter QUV tester (Philips, Saarbrücken, Germany).

### 3.5. Photodegradation of PS Films by IR Spectrophotometer

The FTIR spectra (4000–400 cm^−1^) were recorded on FTIR 8300 Spectrophotometer (Shimadzu, Tokyo, Japan) for the PS films. The carbonyl and hydroxyl group indices (*Is*) can be calculated using Equation (4) [41]. The value of *I*s depends on the peak absorbance (*As*) of C=O or OH group and the reference peak absorbance (*Ar*) at 1328 cm^−1^.

(4) Is=As/Ar

### 3.6. Measuring the Photodegradation by Weight Loss

The weight loss percentage of PS films in photodegradation process was calculated using from the weight of PS sample before (*W*_1_) and after irradiation (*W*_2_) using Equation (5) [42].

(5) $$Weight\;loss\;\%\;=\;\left[\left(W_{1}-W_{2}\right)/W_{1}\right]\;\times \;100$$

### 3.7. Photodegradation of PS Films by Viscometery Method

The average molecular weight (${\bar{M}}_{V}^{\alpha }$) of PS films was measured using Mark–Houwink relation, Equation (6), in which α and *K* are constants [41,43]. ${\bar{M}}_{V}^{\alpha }$ is directly proportional to the intrinsic viscosity, [η], of PS film.

(6) $$\left[\eta \right]=K{\bar{M}}_{V}^{\alpha }$$

Also, the average molecular weight (${\bar{M}}_{V}$) can be calculated using Equation (7).

(7) $$\left[\eta \right]=4.17\;\times \;10^{-4}\;{{\bar{M}}_{V}}^{0.66}$$

## 4. Conclusions

Four Schiff bases with 4*H*-1,2,4-triazole-3-thiol ring systems have been synthesized and characterized. Polystyrene films containing Schiff bases at a concentration of 0.5% were found to more stable on irradiation compared to polystyrene without the additives. Such additives can be used for long-term polystyrene photostabilization. The additives could act as UV absorbers and/or radical scavengers.

## Figures and Tables

**Table 1:** Physical properties and some FT-IR spectral data for Schiff bases **1**–**4**.

Schiff Base	R	Color	m.p. (°C)	Yield (%)	FR-IR (υ, cm^−1^)		
					NH	CH=N	C=S
**1**	Me	yellow	265–268	71	3122	1599	1244
**2**	NO_2_	orange	219–222	79	3257	1589	1240
**3**	OMe	yellow	238–240	68	3120	1606	1244
**4**	NMe_2_	orange	221–224	75	3120	1614	1240

**Table 2:** ^1^H-NMR spectral data for Schiff bases **1**–**4**.

Compound	^1^H-NMR (300 MHz: DMSO-*d*_6_, δ, ppm, *J* in Hz)
**1**	9.56 (s, 1H, CH), 7.84 (d, *J* = 8.1 Hz, 2H, Ar), 7.39 (d, *J* = 8.1 Hz, 2H, Ar), 7.24 (s, 2H, Ar), 3.76 (s, 6H, 2 OMe), 3.74 (s, 3H, OMe), 2.41 (s, 3H, Me)
**2**	9.69 (s, 1H, CH), 8.30 (d, *J* = 8.2 Hz, 2H, Ar), 8.02 (d, *J* = 8.2 Hz, 2H, Ar), 7.20 (s, 2H, Ar), 3.80 (s, 6H, 2 OMe), 3.75 (s, 3H, OMe)
**3**	9.44 (s, 1H, CH), 7.90 (d, *J* = 8.4 Hz, 2H, Ar), 7.23 (s, 2H, Ar), 7.12 (d, *J* = 8.4 Hz, 2H, Ar), 3.85 (s, 3H, OMe), 3.76 (s, 6H, 2OMe), 3.71 (s, 3H, OMe)
**4**	14.09 (s, exch., 1H, SH), 9.13 (s, 1H, CH), 7.73 (d, *J* = 8.2 Hz, 2H, Ar), 7.24 (s, 2H, Ar), 6.81 (d, *J* = 8.2 Hz, 1H, Ar), 3.76 (s, 6H, 2OMe), 3.71 (s, 3H, OMe), 3.04 (s, 6H, NMe_2_)
